# Ewing Sarcoma Protein Ewsr1 Maintains Mitotic Integrity and Proneural Cell Survival in the Zebrafish Embryo

**DOI:** 10.1371/journal.pone.0000979

**Published:** 2007-10-03

**Authors:** Mizuki Azuma, Lisa J. Embree, Hatem Sabaawy, Dennis D. Hickstein

**Affiliations:** Experimental Transplantation and Immunology Branch, Center for Cancer Research, National Cancer Institute, National Institutes of Health, Bethesda, Maryland, United States of America; Max Planck Institute of Molecular Cell Biology and Genetics, Germany

## Abstract

**Background:**

The *Ewing sarcoma breakpoint region 1* gene *(EWSR1)*, also known as *EWS*, is fused to a number of different partner genes as a result of chromosomal translocation in diverse sarcomas. Despite the involvement of EWSR1 in these diverse sarcomas, the *in vivo* function of wild type EWSR1 remains unclear.

**Principal Findings:**

We identified two zebrafish *EWSR1* orthologues, *ewsr1a* and *ewsr1b*, and demonstrate that both genes are expressed maternally, and are expressed ubiquitously throughout zebrafish embryonic development. Morpholino induced knockdown of both zebrafish *ewsr1* genes led to mitotic defects with multipolar or otherwise abnormal mitotic spindles starting from the bud stage (10 hour post-fertilization (hpf)). The abnormalities in mitotic spindles were followed by p53-mediated apoptosis in the developing central nervous system (CNS) leading to a reduction in the number of proneural cells, disorganization of neuronal networks, and embryonic lethality by 5 days post-fertilization. siRNA silencing of EWSR1 in Hela cells resulted in mitotic defects accompanied by apoptotic cell death, indicating that the role of EWSR1 is conserved between zebrafish and human.

**Conclusions:**

Ewsr1 maintains mitotic integrity and proneural cell survival in early zebrafish development.

## Introduction

The *EWSR1* gene is involved in a number of different sarcomas as a result of chromosomal translocations. EWSR1 is fused to an ETS transcription factor (FLI-1, ERG, ETV-1, E1AF or FEV) in Ewing sarcoma; to activating transcription factor-1 (ATF-1) in soft tissue clear cell sarcoma; to Wilms Tumor -1 (WT1) in desmoplastic small round cell; to nuclear receptor 4A3 (NR4A3) in extraskeletal myxoid chondrosarcoma, and to C/EBP-homologous protein (CHOP) in myxoid liposarcoma [1].

Despite the involvement of *EWSR1* fusion genes in tumorgenesis, the function of wild type EWSR1 protein remains unclear. EWSR1 was thought to be involved in transcriptional regulation since it associates with the basal transcription machinery by binding transcription factor IID (TFIID) [2]. In addition, EWSR1 was shown to have transcriptional activity in a cell-specific and promoter-specific manner by interacting with neuronal transcription factors *BRN3-a* and *hepatocyte nuclear factor 4 (HNF4) in vitro* [3], [4]. EWSR1 may also serve as a coupling molecule between transcription and RNA splicing by binding to RNA polymerase II through its N-terminal region and recruiting serine-arginine (SR) splicing factors through its C-terminal domain [5].

Recently, an *EWSR1* gene-targeted mouse was generated which displayed a very high post-natal lethality [6]. Detailed analysis of the few surviving mice demonstrated defects in B lymphocyte maturation, aberrant meiosis, and accelerated senescence of mouse embryonic fibroblasts. The cause of the early post-natal mortality is unclear.

In this report, we analyzed the role of Ewsr1 in early zebrafish embryonic development using morpholino induced knockdown of the two zebrafish Ewsr1 paralogue proteins, designated Ewsr1a and Ewsr1b. Zebrafish is particularly suitable for analysis of early embryonic development due to their transparent embryos which develop externally. Here, we demonstrate that the zebrafish *ewsr1* genes are required for mitotic integrity and survival of neural cells in the zebrafish CNS during early embryonic development, and that the involvement of EWSR1 in mitotic stability is conserved from zebrafish to human.

## Results

### Maternal and ubiquitous expression of *ewsr1a* and *ewsr1b* mRNAs in zebrafish embryos

**Figure 1:**
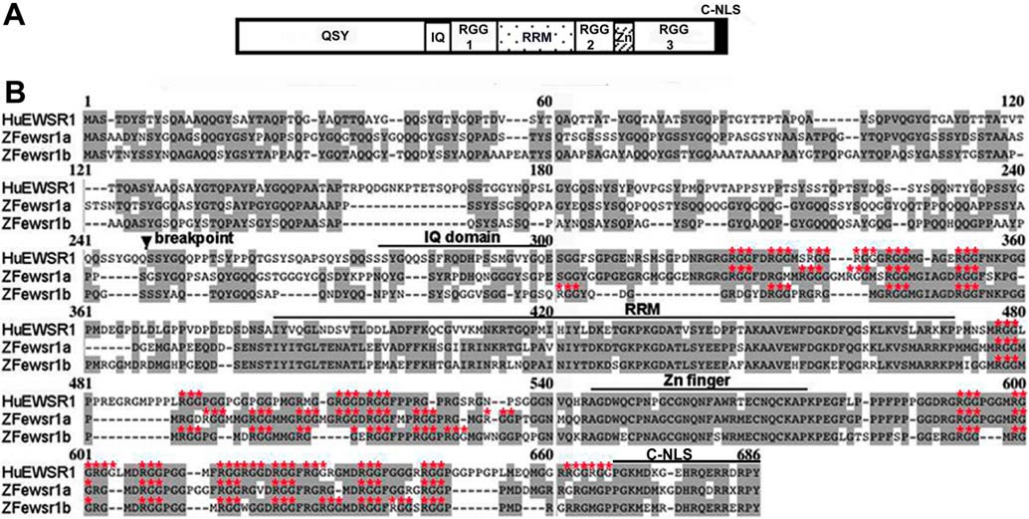
Sequence Analysis of Zebrafish *ewsr1* Genes Indicates Homology in Putative Functional Domains. A. Schematic drawing of the human EWSR1 protein structure. Break point: breakpoint of Ewing sarcoma (type I), QSY: QSY rich domain, IQ: IQ domain, RGG: RGG box, RRM: RNA Recognition Motif, Zn: Zn finger domain and C-NLS: C-terminus nuclear localization signal. B. Predicted protein sequence of human EWSR1 and zebrafish Ewsr1a and Ewsr1b. The identical amino acids between human (Hu), and zebrafish (ZF) proteins are boxed, domains are underlined and the RGG repeats are marked with asterisks (*).

We identified two genes, designated *ewsr1a* and *ewsr1b*, displaying homology to human *EWSR1*, also known as *EWS*. Zebrafish Ewsr1a predicted a 626 amino acid protein, and Ewsr1b had a predicted length of 579 amino acids (Figure 1B). The amino acid sequence identity between human EWSR1 and the zebrafish proteins was more conserved in the C-terminal RNA binding motifs (RGG), the RNA recognition domain (RRM), the zinc finger domain (Zn) and C-terminal nuclear localization signal (C-NLS), compared to the N-terminus region (Figure 1A and B) [7]. It is noteworthy that these regions are not present in the expressed fusion genes found in sarcomas since these domains are located c-terminal of the chromosomal breakpoint (Figure 1B). The amino acid identity and the phylogenic tree for the EWSR1 proteins demonstrated the Ewsr1a and Ewsr1b have highest identity to the human and mouse EWSR1, rather than other members of the TLS/EWS/TAF15 (TET) gene family (Figure S1).

**Figure 2:**
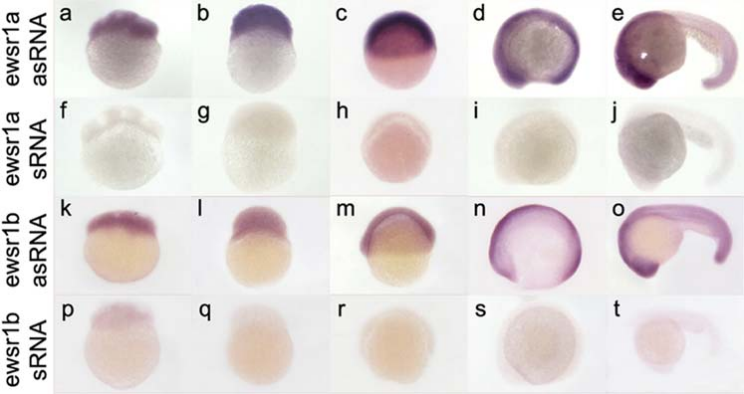
Initial Maternal and Subsequent Ubiquitious Expression of *ewsr1a* and *ewsr1b* mRNAs in Zebrafish Embryonic Development. The images are lateral views of embryos stained by *in situ* hybridization using anti-sense RNA (a–e, k-o) or sense RNA (f–j, p–t) for *ewsr1a* and *ewsr1b*. The *ewsr1a* and *ewsr1b* were expressed ubiquitously at 4 cell (a and k), 256 cell (b and l), shield (c and m), 5 somite stage (d and s) and 24hpf (e and t). asRNA: anti-sense RNA, sRNA: sense RNA

Human *EWSR1* mRNA expression is ubiquitous in adult tissues [8]. However, there is no description of *EWSR1* mRNA expression during embryonic development. We determined the spatial and temporal expression patterns of zebrafish *ewsr1a* and *ewsr1b* during development using whole-mount *in situ* hybridization. Both *ewsr1a* and *ewsr1b* were maternally expressed as early as the one cell stage (data not shown), and both genes continue to be expressed ubiquitously throughout early embryonic development (Figure 2, a∼e and k∼o).

### Morphological defects in the CNS in *ewsr1a* and *ewsr1b* deficient zebrafish embryos are accompanied by apoptotic cell death

**Figure 3:**
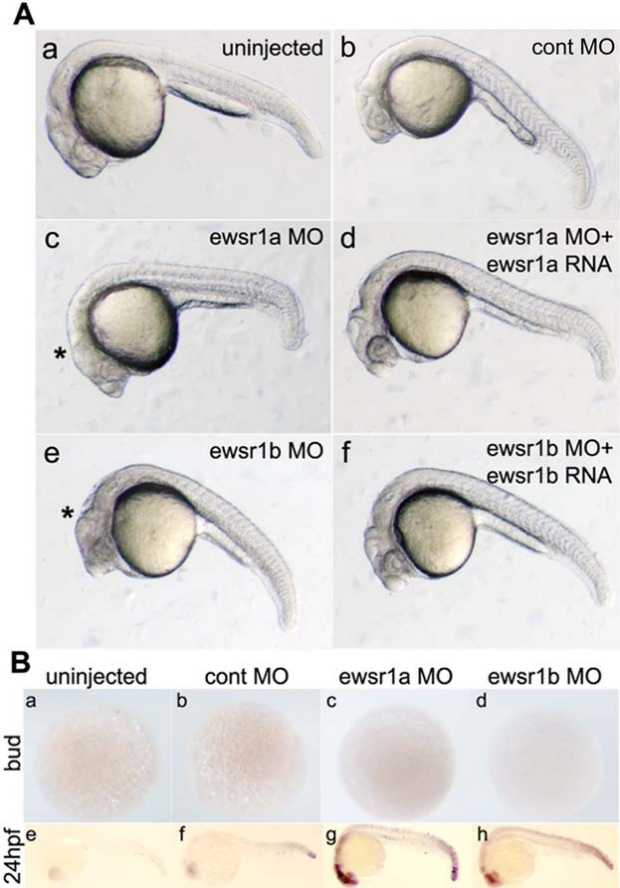
MO-Induced Knockdown of Zebrafish *ewsr1a* and *ewsr1b* Results in Morphologic Abnormalities in the CNS Accompanied by Apoptosis. A. (a) uninjected, (b) control morpholino (MO), (c) *ewsr1a* MO , (d) *ewsr1a* MO with *ewsr1a* mRNA , (e) *ewsr1b* MO, and (f) *ewsr1b* MO and *ewsr1b* mRNA co-injected embryo. Both *ewsr1a* and *ewsr1b* MO injected embryos show dark cells (c and e) in the brain. The asterisk (*) indicates increased apoptosis and abnormal MHB. Co-injection of MO and mRNA rescued normal brain morphology (d and f). All of the images are lateral views of 24hpf zebrafish embryos. B. No increase of apoptosis in the bud stage (a–d), increased apoptosis in the CNS of *ewsr1a* and *ewsr1b* -MO injected embryos at 24hpf (g and h compared to e and f). Lateral views of wild type (a and e), control MO injected embryo (b and f), *ewsr1a* MO injected embryo (c and j) and *ewsr1b* MO injected embryo (d and h), after TUNEL staining.

To determine the role of Ewsr1a and Ewsr1b in zebrafish embryonic development, morpholinos (MO) directed against the two ewsr1 mRNAs were injected into zebrafish embryos at the one cell stage. MO-induced knockdown of *ewsr1a* or *ewsr1b* did not result in morphologic abnormalities during the gastrulation period. However, starting with the bud stage at 10 hours post-fertilization (10 hpf), development was delayed, and dark cells began appearing in the entire brain by the mid-somitogenesis stage (data not shown). A consistent phenotype involving abnormalities of the midbrain-hindbrain boundary (MHB) became apparent at 24 hpf (Figure 3A, c and e). Injection of the control MO did not affect the morphology (Figure 3A, b). Co-injection of *ewsr1a* mRNA with the *ewsr1a* MO or *ewsr1b* mRNA with the *ewsr1b* MO rescued the morphological defect caused by MO injection, indicating the specificity of the effect of the *ewsr1a* and *ewsr1b* MO (Figure 3A, panels d and f). Scores for the zebrafish embryonic abnormalities resulting from injection of MO are listed (Table S1).

To examine whether the morphological changes in the *ewsr1a* and *ewsr1b* deficient zebrafish CNS were due to apoptotic cell death, the TUNEL assay was performed. There were very low levels of apoptotic cell death in the wild type and control MO injected embryos (Figure 3B, a, b, e, f) [9]. There was no apparent increase of TUNEL positive cells in the bud stage (10 hpf) of the *ewsr1a* and *ewsr1b* MO injected embryos (Figure 3B, c and d). However, there was a marked increase in apoptotic cell death in the brain and spinal cord starting around the mid-somite stages. The number of apoptotic cells in the brain and spinal cord in the MO injected embryos increased further at 24 hpf (Figure 3B, compare g and h to e and f). These results indicated that the *ewsr1* genes are required for cell survival in the developing CNS.

### Reduction of proneural cells and disorganization of the neuronal networks in *ewsr1a* and *ewsr1b* knockdown embryos

**Figure 4:**
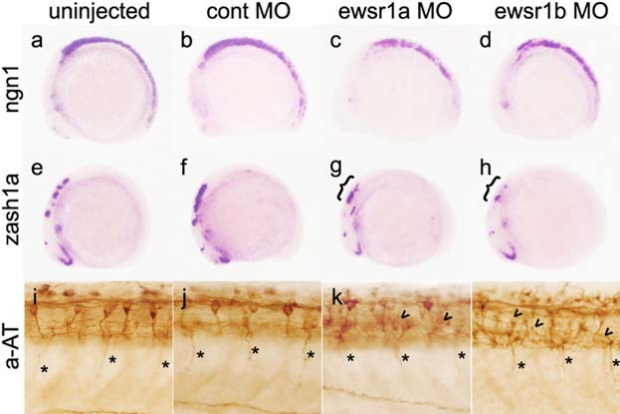
*ewsr1* MO Injected Embryos Display a Reduction of CNS Neuronal cells and Disorganized Neuronal Network. (a, e and i) uninjected, (b, f and j) control MO, (c, g and k) *ewsr1a* MO and (d, h and l) *ewsr1b* MO injected embryo. (a–d) *ewsr1a* and *ewr1b* MO injected 12 somite embryos show marked reduction of *ngn1* expression in the CNS (lateral view). (e–h) *ewsr1a* and *ewr1b* MO injected 12 somite embryos show marked reduction of *zash1a* expression in the CNS (lateral view). ({) indicate marked reduction in the hindbrain of *zash1a*. (i–l) Lateral views of the trunk in 24hpf embryos stained with acetylated tubulin (aAT) antibody. Arrowhead identifies disorganized axonal projections in *ewsr1* MO injected embryos. Asterisk (*) marks motorneurons.

To determine the cell types affected in the MO injected embryos, we performed *in situ* hybridization using marker genes. Expression of bHLH proneural specific transcription factor genes, *zash1a* and *ngn1,* was significantly reduced in the brain in *ewsr1a* and *ewsr1b* MO injected embryos at the 12 somite stage (Figure 4, c, d, g, and h) compared to uninjected and control MO injected embryos (Figure 4, a, b, e, and f) [10], [11], [12]. Other markers, including the hindbrain marker *krox20*, paraxial mesoderm marker *ntl*, and notocord marker *shh*, were not affected in the MO injected embryos, suggesting that the overall patterning is not affected (Figure S2) [13], [14], [15]. The neuronal network was examined by acetylated tubulin antibody staining and demonstrated extreme disorganization of the spinal cord in the *ewsr1a* and *ewsr1b* MO injected embryos (Figure 4 k and l). These studies indicate that neuronal differentiation is perturbed and the neuronal network is disorganized in *ewsr1a* and *ewsr1b* deficient zebrafish embryos.

### P53-mediated apoptotic cell death in the CNS of the *ewsr1a* and *ewsr1b* deficient zebrafish embryos

**Figure 5:**
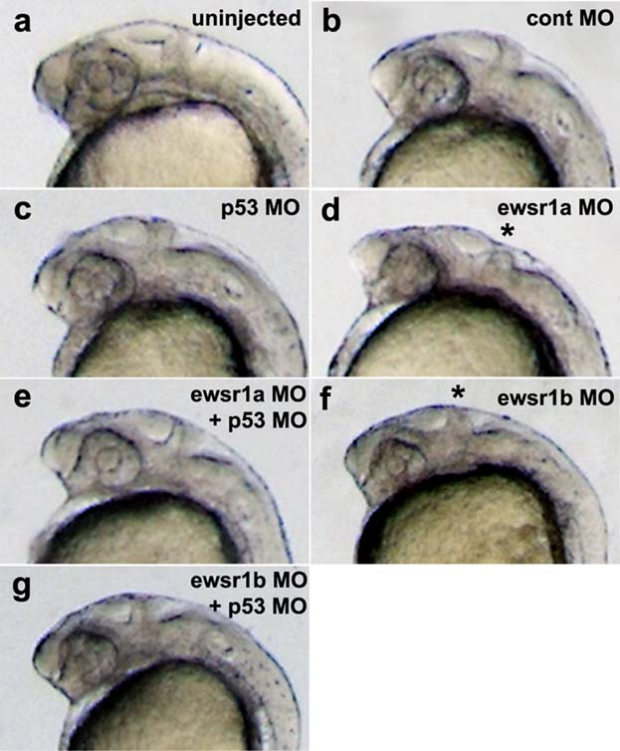
MO Knockdown of p53 Rescues Morphologic Abnormalities in ewsr1 Knockdown Embryos. Lateral views of (a) uninjected, (b) control MO, (c) control MO and *p53* MO, (d) *ewsr1a* MO, (d) *ewsr1a* MO+*p53* MO injected embryo, (e) *ewsr1b* MO and (f) *ewsr1b* MO+*p53* MO injected embryo at 24hpf. Asterisk (*) indicates the normal MHB structure (d and f).

To assess the involvement of P53 in the induction of apoptosis, *ewsr1a* or *ewsr1b* MO were co-injected along with a *p53* MO. Neither control MO alone, nor control MO plus *p53* MO, altered the development of the CNS in the zebrafish embryos (Figure 5, b and c). However, when the *p53* MO was co-injected with the *ewsr1a* MO or the *ewsr1b* MO, the morphological defect seen in the brain with the *ewsr1a* or *ewsr1b* MO alone was rescued, indicating that the morphological defects in the CNS induced by knock-down of *ewsr1a* and *ewsr1b* were mediated by the P53 pathway (Figure 5, compare d to e and f to g). Scoring of morphology from the uninjected and MO injected embryos is listed in Table S2.

### Loss of mitotic integrity in the *ewsr1a* and *ewsr1b* deficient zebrafish embryos

**Figure 6:**
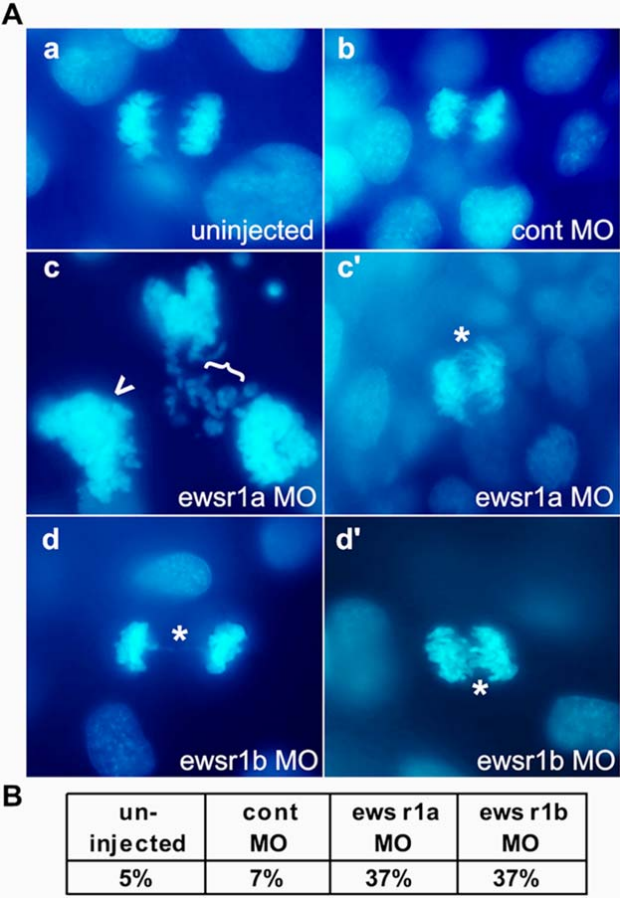
* ewsr1* MO Injected Zebrafish Embryos Display Genomic Instability. A. Representative images of mitotic spindles from (a) uninjected, (b) control MO, (c and c') *ewsr1a* MO and (d and d') *ewsr1b* MO injected embryo. DNA was stained with DAPI (blue). Asterisks (*) identify chromosome bridges. (c) The cell appears to be the result of aberrant chromosomal segregation. ({) points to missegregated chromosomes and arrowhead (<) to a possible additional chromosome set. B. Percentage of chromosome bridges per hundred anaphase chromosomes of uninjected, control MO, *ewsr1a* MO and *ewsr1b* MO injected embryos at the 12 somite stage (12sm).

During the analysis of the phenotype of the *ewsr1a* and *ewsr1b* deficient embryos, we observed a number of abnormal condensed chromosomes accompanied by chromosomal bridges (compare Figure 6A a, b to c, c', d and d'). To assess genomic instability, we observed one hundred anaphase spindles in 12 somite stage *ewsr1a* and *ewsr1b* knockdown embryos, and the percentage of cells displaying chromosomal bridges was scored. The scores were significantly higher in *ewsr1a* and *ewsr1b* knockdown embryos compared to uninjected or control MO injected embryos (Figure 6B), indicating higher genomic instability in the *ewsr1a* and *ewsr1b* knockdown embryos.

**Figure 7:**
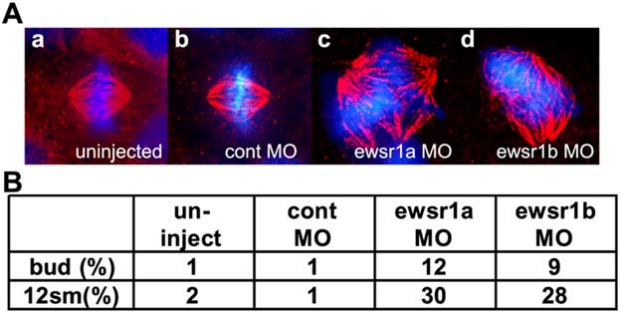
* ewsr1* MO Injected Zebrafish Embryos Display Mitotic Defects. A. Representative images of mitotic spindles from (a) uninjected, (b) control MO, (c) *ewsr1a* MO and (d) *ewsr1b* MO injected embryos. DNA was stained with DAPI (blue) and the spindles were visualized by α-tubulin staining (red). B. Score of abnormal mitotic spindles of uninjected, control MO, *ewsr1a* MO and *ewsr1b* MO injected embryos at the bud and the 12 somite stages (12sm). Approximately two hundred mitotic spindles (N = 193–234) were examined for each group.

Chromosomal bridging can be caused by mitotic defect, therefore mitotic spindles were examined at the bud stage (10 hpf) and at the 12 somite stage (16 hpf) in early *ewsr1* gene depleted zebrafish embryos. These stages were selected since the TUNEL assay indicated that there was no increase in apoptotic cell death in the bud stage, with apoptosis starting to increase at the mid-somite stage. Mitotic spindles were visualized with *α*-tubulin antibody and DNA with DAPI. For each stage, spindles were scored for any abnormalities, including disorganized structure, multipolar or monopolar spindles (Figure 7A). Approximately two hundred mitotic cells derived from twenty embryos were scored from each sample group (Figure 7B). Uninjected and control-MO injected embryos showed a very low incidence of abnormal spindles (1–2%). In contrast, a significant increase in mitotic defects was observed starting from the bud stage in *ewsr1a* and *ewsr1b* knockdown embryos (Figure 7B). Moreover, the incidence of abnormal spindles increased markedly at the 12 somite stage in the *ewsr1* knockdown embryos compared to the bud stage, indicating the accumulation of mitotic defects during development. It is noteworthy that we observed increased numbers of mitotic defects in the bud stage, before apoptosis developed, indicating that the mitotic spindle defects preceded apoptotic cell death.

### Loss of mitotic integrity accompanied by mislocalization of Aurora B proteins in EWSR1 deficient Hela cells

**Figure 8:**
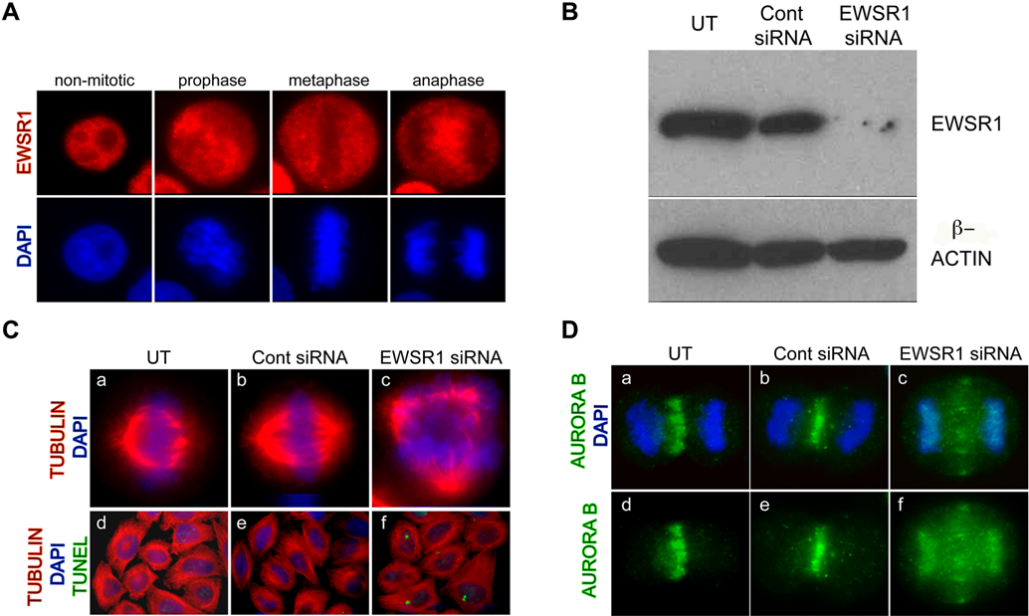
EWSR1 siRNA transfected Hela cells Display Mitotic Defects. A. EWSR1 localization in Hela cells during non-mitotic and mitotic-stages. EWSR1 was visualized by anti-EWSR1 antibody (red) and DNA was stained with DAPI (blue). B. EWSR1 protein (top panel) and β-Actin (bottom panel) visualized by western blotting. C. Representative images of mitotic spindles from (a and d) UT, (b and e) Cont siRNA and (c and f) EWSR1 siRNA transfected Hela cells. DNA was stained with DAPI (blue), the spindles were visualized by α-Tubulin (red) and the apoptotic cells were visualized by TUNEL assay (green). D. Representative images of Aurora B (green) with DNA (blue) (a–c), and Aurora B (green) without DNA (d–f). (a and d) UT, (b and e) Cont siRNA and (c and f) EWSR1 siRNA transfected Hela cells. UT: untransfected, Cont siRNA: control siRNA transfected, EWSR1 siRNA: EWSR1 siRNA transfected Hela cells.

The Ewsr1 knockdown in zebrafish embryos induced mitotic defects accompanied by apoptotic cell death. To determine whether this effect was conserved between zebrafish and human cells, and to address this phenotype at the molecular level, we investigated the role of EWSR1 in Hela cells. These studies were facilitated by a number of antibodies to the human mitotic components. EWSR1 localized to the nucleus in non-mitotic Hela cells, and to the entire cell during mitosis when the nuclear envelope breaks down (Figure 8 A). This observation is consistent with a role for EWSR1 in mitosis. To address the detailed molecular events in mitosis, we utilized EWSR1 siRNA. Hela cells were transfected with EWSR1 siRNA or control siRNA and analyzed by western blotting. The EWSR1 siRNA transfected cells exhibited marked reduction of the EWSR1 protein level compared to cells transfected with control siRNA or untransfected cells (Figure 8 B, top panel). To determine whether the mitotic defect phenotype observed in Ewsr1 knockdown zebrafish embryos was conserved in human cells, mitotic spindles in Hela cells were visualized by anti-α tubulin staining. The EWSR1 siRNA transfected Hela cells exhibited a significantly higher incidence of abnormal spindles (30%), including multipolar spindles, compared to untransfected (4%) and control siRNA transfected Hela cells (4%) (N>50 mitotic cells). Representative images of mitotic spindles are shown (Figure 8 C, compare c to a and b). This phenotype was consistent with the result observed in Ewsr1 knockdown zebrafish embryos, indicating that EWSR1 is required for mitotic integrity in both species. In addition, increased apoptosis was observed in the EWSR1 siRNA transfected cells (22%) compared to the untransfected (1%) and control siRNA transfected cells (3%) by TUNEL assay, indicating that EWSR1 maintains cell survival (N>100 TUNEL positive cells) (Figure 8 C, compare f to d and e).

To investigate the relationship between the mitotic spindle abnormalities and EWSR1, we assessed Aurora B kinase, as it is a component of the chromosome passenger complex (CPC) known to play a major role in mitotic checkpoints [16], [17]. Localization of the CPC complex is tightly regulated. In early mitotsis, this complex associates with the inner centromere, transfers to the central spindle in anaphase, and associates with the midbody during cytokinesis [16]. Aurora B is localized at the central spindle during anaphase (Figure 8 D a, b, d and e). However, in EWSR1 siRNA transfected cells, Aurora B localized at the area between chromosomes and central spindle (Figure 8 D c and f), indicating a mislocalization of the CPC complex and potential loss of its checkpoint function. The percentage of cells that exhibit mislocalization of Aurora B in EWSR1 siRNA transfected cells was 29%, untransfected cells was 0% and control siRNA transfected cells was 7% (N>50 anaphase cells). These results indicate that EWSR1 is required for proper localization of Aurora B during mitosis.

## Discussion

The zebrafish represents a powerful vertebrate model for the analysis of the earliest stages of embryonic development, including gastrulation, somitogenesis and brain formation [18]. This is especially important for analysis of *ewsr1* gene function since gene targeting of EWSR1 in the mouse resulted in an unexplained early post-natal lethality [6]. To analyze the role of the *ewsr1* gene *in vivo* during zebrafish embryonic development, we isolated two *ewsr1* zebrafish homologs, *ewsr1a* and *ewsr1b,* and demonstrated that both zebrafish *ewsr1* genes were expressed maternally, and continued to be expressed ubiquitously during early development. MO knockdown of either *ewsr1* gene led to P53-mediated apoptosis in the brain and the spinal cord at the mid-somite stage, indicating that the Ewsr1 proteins are required for cell survival in the CNS. There were increased numbers of aberrant mitotic spindles prior to the apoptotic cell death, suggesting that the *ewsr1* genes are necessary for proper mitotic spindle integrity, which in turn is required for cell survival.

Despite ubiquitous expression of *ewsr1a* and *ewsr1b* at the mRNA level, MO knockdown experiments of each zebrafish *ewsr1* gene leads to defects in the brain and spinal cord. However, silencing of EWSR1 in Hela cells leads to mitotic defects, suggesting that the mitotic defect caused by silencing of EWSR1 is not limited to the CNS cells. It is possible that apoptosis occurs first in CNS cells followed by non-CNS cells. It is unknown why the CNS cells are prone to apoptosis by Ewsr1 knockdown. One possibility may be the interaction between the Ewsr1 and a neuronal specific factor. In this regard, EWSR1 has been reported to activate the promoter of the neuronal specific POU transcription factor Brn-3a through the formation of a heterodimer [3], [19], [20]. Brn-3a regulates neuronal outgrowth and prevents apoptotic cell death of sensory neurons by activating the promoters of multiple factors, including Trk A receptors and Bcl-2 [19], [21], [22]. Trk A is a tyrosine kinase receptor that is stimulated by NGF, and prevents apoptosis and regulates differentiation in neurons [23]. Bcl-2 is also required for cell survival. It is possible that *ewsr1* knockdown prevents activation of the *Brn-3a* promoter, causing a reduction of the target genes Trk A and Bcl-2, which results in apoptotic cell death [24].

An alternative explanation for the apoptotic cell death in the CNS is that *ewsr1* knockdown may lead to disorientation of the spindle resulting in aberrant cell alignment. This altered cell alignment may cause differentiation defects in neuronal tissues. During the gastrula and neurula stages in zebrafish embryos, the orientation of cell division is tightly regulated [25]. At these stages, the embryo is dramatically increasing its size through increasing cell number, and is organizing these cells by dynamic cell movements. The orientation of the cell division plane defines the location of daughter cells after each cell division, and this orientation is determined by the orientation of the spindles. For example, dividing cells are oriented in parallel to the neuroepithelium plane, while cell divisions in the neural keel are perpendicular due to a 90° rotation of the mitotic spindle [26]. In Ewsr1 knockdown embryos, we observed an increased number of aberrant spindles including aneuploidy, tetraploidy and abnormal spindle structure at the bud stage, a time immediately after the three germ cell layers are specified. The aberrant spindle assembly may also affect the position of the daughter cells, and ultimately affect its cell organization and differentiation in tissues. Thus, it is possible that the disorganization of the neuronal network in the *ewsr1* knockdown embryos is derived from disorganized spindles.

We demonstrated mislocalization of the Aurora B kinase in EWSR1 siRNA transfected Hela cells. Aurora B is one of the components of the CPC complex that plays a major role in checkpoint control in mitosis [16]. Mislocalization of Aurora B may lead to defects in checkpoint function during mitosis. Currently, it is unknown whether EWSR1 is influencing Aurora B localization directly or indirectly. It is possible that the mitotic defect is triggered by DNA pairing or repair/recombination defects in EWSR1 deficient cells as reported previously [6]. In future studies, it will be important to examine the influence of EWSR1 on Aurora B localization at the molecular level.

In EWSR1 fusion gene expressing sarcomas, one *EWSR1* allele is disrupted as a result of chromosomal translocation. If this loss results in haploinsufficiency, this may lead to mitotic defects and genomic instability such as we have demonstrated in zebrafish embryos and human cells. Thus, these observations may be important for understanding the function of EWSR1 during development as well as the potential pathogenesis of *EWSR1* fusion gene expressing sarcomas.

## Materials and Methods

### Isolation of zebrafish *ewsr1* cDNAs

A tBlastn search using the NCBI database identified the zebrafish EST clones Agencourt 1691712 and fc04c01, which we designated *ewsr1a*, and TC237536, which we designated *ewsr1b*. Although TC237536 was described as a FUS homolog in the database, it showed higher homology to EWSR1 (52% identity; Figure S1, A) compared to FUS. Therefore, we designated this clone as a zebrafish homolog of human *EWSR1* gene. The full-length cDNA clones were amplified by PCR from cDNA isolated from 24 hours post fertilization (hpf) whole embryos using the following primers: for *ewsr1a* (5′-TCAGGGTACCATGGCACACGAAATGGC-3′) and (5′-ATACTGGTTTGGTGGTTTATAG-3′), and for *ewsr1b* (5′–TCAGGGTACCATGGCGTCAGTCACGAA-3′) and (5′-ATAAGGATTTTGCTGGTAATC-3′). For first strand synthesis, 1 µg of total RNA was mixed with the primers, 2.5 uM oligo dT, 500 uM of dNTP, 5 mM DTT and 10 U of MMLV reverse transcriptase (Roche) in 20 µl. The amplified cDNAs were cloned into a TA-cloning vector (pGEM-T easy vector, Promega) and sequenced.

### In situ hybridization

*In situ* hybridization was performed as described previously [27], [28].

### Morpholino and synthetic RNA injection

Morpholinos were obtained from Gene Tools, LLC, with the following sequences: Control MO, 5′-CCTCTTACCTCAGTTACAATTTATA-3′, *ewsr1a* MO, 5′- AGACGCCATTTCGTGTGCCATCCCG-3′: *ewsr1b* MO, 5′-GCTATAATTCGTGACTGACGCCATC-3′. Both *ewsr1a* and *ewsr1b* MO sequences include the predicted ATG start codon. The sequence of the p53 MO was designed as previously reported [29]. In all studies, 1 nl of 10 ng/nl control or *ewsr1b* MO, 5 ng/nl of *ewsr1a* MO or 3 ng/nl of p53 MO were injected into 1 cell stage embryos. For rescue experiments, the rescue DNA constructs that contain Kozac consensus sequence and five silent mutations were generated by amplifying the full-lenfth *ewsr1* cDNAs as a template for a PCR reaction at the *Hind*III site to *Bpi*I site for *ewr1a* and *Hind*III site to *Sda*I for *ewsr1b*. Digested fragments (*Hind*III/*Bpi*I fragment for *ewsr1a* and *Hind*III/*Sda*I fragment for *ewsr1b*) were ligated to the *Hind* III/*Bpi* I fragment from *ewsr1a* full length cDNA and *Hind* III/*Sda* I fragment from *ewsr1b* full length cDNA. The primer sequences are: *ewsr1a* (5′-AGGCAAGCTTGCCACCATGGCGCATGAGATGGCATCC-3′) and (5′-GATCGAAGACCCATATGGCTGC-3′), *ewsr1b* (5′-AGGCAAGCTTGCCACCATGGCCTCGGTTACAAAC-3′) and (5′-GATCCCTGCAGGCTGCGAATAG-3′). *ewsr1a* and *ewsr1b* mRNAs were synthesized (Message Machine; Ambion). 1 nl of 400 pg/nl of the *ewsr1a* mRNA and 600 pg/nl of *ewsr1b* mRNA solutions were injected into zebrafish embryos, respectively.

### TUNEL assay

TUNEL assays in zebrafish embryo were performed as described previously [30]. TUNEL assays in Hela cells were performed using a TUNEL assay kit (Roche) as described in the manufactor's protocol.

### Immunohistochemistry

The mitotic spindles were visualized by immunohistochemistry using an α-tubulin antibody as described previously with minor modifications [31]. In this study, mouse monoclonal α-tubulin antibody (SIGMA, MO) was used at a dilution of 1:4000, and mouse Alexa 594 was used as a secondary antibody at 1:250 in the same blocking solution as described. The yolk was removed, and the embryos were mounted on glass slide with DAPI/Vectashield (Molecular probes). The images are the maximum z-projection of cells taken with an UltraVIEW spinning disc confocal microscope (Perkin Elmer).

For acetylated tubulin antibody staining, embryos were fixed with 4% paraformaldehyde (PFA) overnight at 4°C. The fixed embryos were washed twice with PBS and fixed in 100% methanol overnight at −20°C. The embryos were then rinsed twice in PBS, digested with 0.25% trypsin on ice for 5 min, and washed in 0.8% Triton X-100/PBS solution for 5 min, five times. The embryos were treated with acetone for 7 min at −20°C. After washing with 0.8% Triton X-100/PBS solution for 5 min three times, the embryos were blocked with solution A (10% FCS, 1% DMSO, 0.1% Triton X-100 in PBS) for 1 hr at room temperature. The embryos were treated with anti-acetylated tubulin antibody (SIGMA) at a dilution of 1:5000 in solution A overnight at 4°C. After washing with solution A for 30 min five times at room temperature, the embryos were incubated overnight at 4°C in mouse secondary antibody at a dilution of 1:2500. The embryos were then washed with solution B (1% DMSO, 0.1% Triton X-100 in PBS) 3 times for 30 min. The embryos were incubated in avidin-biotin at a dilution of 1:300 (Vector Laboratories) for 2 hrs at room temperature, then washed with PBS, and the signals were visualized with 0.1 mg/ml DAB in 0.006% H2O2 in PBS.

### Immunocytochemistry

Twenty four hours after transfection of the siRNA, cells were fixed with 4% paraformaldehyde (PFA) for 1 hr at 4°C. The fixed cells were washed twice with PBS permeabilized with methanol/acetone (1∶1 mixture) for 10min at −20°C. The cells were dried, rinsed twice with PBS and blocked with blocking solution (1% FBS in PBS) 1 hr at room temperature. The cells were rinsed three times with PBS and treated with first antibody solution. Anti-EWSR1 antibody (ABR Affinity BioReagents, CO) was used at a dilution of 1∶1000, and anti α-tubulin antibody (SIGMA, MO) at a dilution of 1∶4000. The cells were rinsed three times with PBS and treated with secondary antibody Alexa 594 (INVITROGEN, CA) at a dilution of 1∶250 for 1 hr at room temperature. The cells were rinsed three times with PBS and were mounted on glass slides with DAPI/Vectashield (INVITROGEN, CA). The images were taken with a PROVIS AX70 microscope (OLYMPUS).

### Gene silencing using siRNA

Double stranded siRNA for EWSR1 silencing and the control siRNA were purchased from Santa Cruz Biotechnology, Inc. Hela cells were plated in 6 well plates and transfected with 70 pmol of EWS siRNA or control siRNA in 1 ml of medium as described in the manufactor's protocol (Santa Cruz Biotechnology, Inc., CA). Cells were harvested 24 hr after transfection and analyzed by western blotting or immunocytochemistry.

### Western blotting

Hela cells were lysed 24 hour after transfection with 1× SDS buffer and subjected to western blotting following a standard protocol using anti-EWS antibody at a dilution of 1∶1000 (Affinity BioReagents, CO).

## Supporting Information

Figure S1 Sequence analysis of Ewsr1 proteins. (A) Phylogenic tree based on full-length amino acid sequences. (B) The numbers represent the percentage of amino acid identity among species. Hu: human, Mo: mouse and ZF: zebrafish.
(0.31 MB TIF)

Figure S2 Lateral views of krox20, ntl and shh Embryos Demonstrate Normal Hindbrain, Axial Mesoderm, and Notochord Patterning. (a, e and i) uninjected, (b, f and j) control MO, (c, g and k) ewsr1a MO, and (d, h and l) ewsr1b MO injected embryo.
(0.41 MB DOC)

Table S1 Phenotype of Uninjected, Control MO, *ewsr1a* MO, *ewsr1a* MO+*ewsr1a* mRNA, *ewsr1b* MO, and *ewsr1b*+*ewsr1b* mRNA Injected Embryos. Number of atypical phenotypes: a = 9, b = 7, c = 5, d = 4 and e = 11.
(0.02 MB DOC)

Table S2 Phenotype of Uninjected, Control MO, *ewsr1a* MO, *ewsr1a* MO+*p53* MO, *ewsr1b* MO, *ewsr1b* MO+*p53* MO Injected Embryos. Number of atypical phenotypes: a = 2, b = 8, c = 2 and d = 3.
(0.02 MB DOC)
