# Supplementary figures and images for: Ewing Sarcoma Protein Ewsr1 Maintains Mitotic Integrity and Proneural Cell Survival in the Zebrafish Embryo
Source: PLoS One. 2007 Oct 3;2(10):e979. doi: 10.1371/journal.pone.0000979 (PMC1991596; doi:10.1371/journal.pone.0000979)

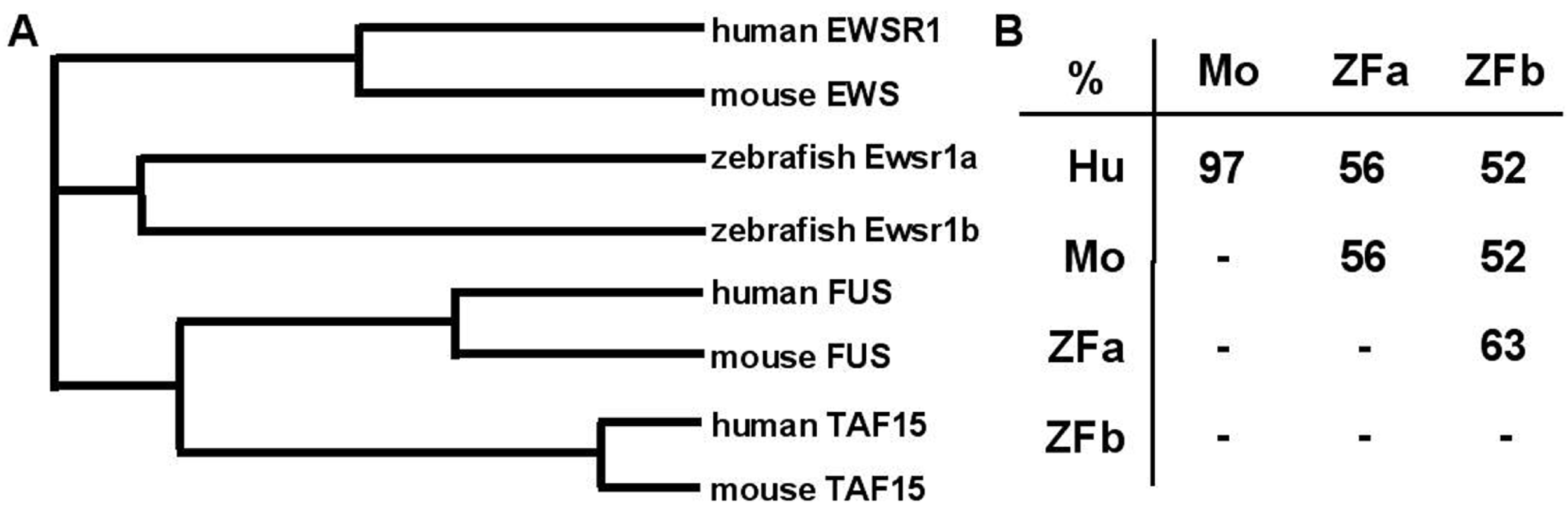

Supplement: Figure S1 — Sequence analysis of Ewsr1 proteins. (A) Phylogenic tree based on full-length amino acid sequences. (B) The numbers represent the percentage of amino acid identity among species. Hu: human, Mo: mouse and ZF: zebrafish. (0.31 MB TIF) [file pone.0000979.s001.tif]

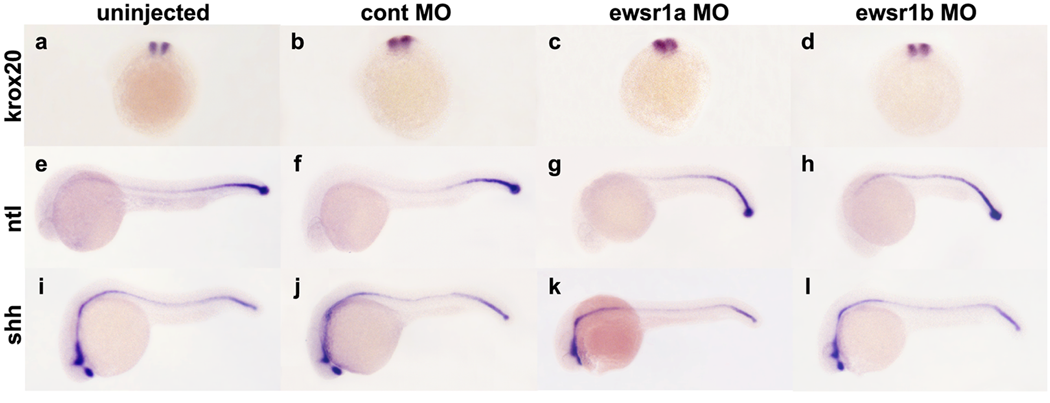

Supplement: Figure S2 — Lateral views of krox20, ntl and shh Embryos Demonstrate Normal Hindbrain, Axial Mesoderm, and Notochord Patterning. (a, e and i) uninjected, (b, f and j) control MO, (c, g and k) ewsr1a MO, and (d, h and l) ewsr1b MO injected embryo. (0.41 MB DOC) [file pone.0000979.s002.doc]
