# Supplementary material for: Ewing Sarcoma Protein Ewsr1 Maintains Mitotic Integrity and Proneural Cell Survival in the Zebrafish Embryo
Source: PLoS One. 2007 Oct 3;2(10):e979. doi: 10.1371/journal.pone.0000979 (PMC1991596; doi:10.1371/journal.pone.0000979)
Supplement: Table S1 — Phenotype of Uninjected, Control MO, ewsr1a MO, ewsr1a MO+ewsr1a mRNA, ewsr1b MO, and ewsr1b+ewsr1b mRNA Injected Embryos. Number of atypical phenotypes: a = 9, b = 7, c = 5, d = 4 and e = 11. (0.02 MB DOC) [file pone.0000979.s003.doc]

### Table S1

|  | normal %  (no) | abnormal %  (no) |
| --- | --- | --- |
| uninjected | 89  (79) | 11^a^  (9) |
| cont MO | 86  (43) | 14^b^  (7) |
| *ewsr1a* MO | 9  (5) | 91^c^  (48) |
| *ewsr1a* MO+ *ewsr1a* RNA | 29  (20) | 71^d^  (49) |
| *ewsr1b* MO | 6  (2) | 94  (34) |
| *ewsr1b* MO+ *ewsr1b* RNA | 29  (14) | 71^e^  (35) |
