# Supplementary material for: Ewing Sarcoma Protein Ewsr1 Maintains Mitotic Integrity and Proneural Cell Survival in the Zebrafish Embryo
Source: PLoS One. 2007 Oct 3;2(10):e979. doi: 10.1371/journal.pone.0000979 (PMC1991596; doi:10.1371/journal.pone.0000979)
Supplement: Table S2 — Phenotype of Uninjected, Control MO, ewsr1a MO, ewsr1a MO+p53 MO, ewsr1b MO, ewsr1b MO+p53 MO Injected Embryos. Number of atypical phenotypes: a = 2, b = 8, c = 2 and d = 3. (0.02 MB DOC) [file pone.0000979.s004.doc]

Table S2

|  | normal %  (no) | abnormal %  (no) |
| --- | --- | --- |
| uninjected | 100  (92) | 0  (0) |
| cont MO | 92  (24) | 8^a^  (2) |
| *P53* MO | 83  (41) | 17^b^  (8) |
| *ewsr1a* MO | 2  (1) | 98  (63) |
| *ewsr1a* MO  + *p53* MO | 48  (0) | 52^c^  (23) |
| *ewsr1b* MO | 8  (3) | 92^d^  (36) |
| *ewsr1b* MO  + *p53* MO | 60  (21) | 40  (14) |
